# Exogenous melatonin enhances salt tolerance in *Pyrus calleryana* through coordinated regulation of osmotic adjustment and antioxidant defense

**DOI:** 10.1186/s12870-026-09137-x

**Published:** 2026-06-04

**Authors:** Yuze Liu, Bin Lu, Xinyue Yu, Mingyuan Hu, Zhaoxi Wang, Xiaoyue Yu

**Affiliations:** 1 College of Forestry, Hebei Agricultural University, Baoding, China; 2 Hebei Key Laboratory of Floral Biological Breeding, Baoding, 071000 China; 3 Hebei Key Laboratory for Tree Genetic Resources and Forest Protection, Baoding, 071000 China

**Keywords:** Salinity tolerance, Melatonin, Reactive oxygen species, Osmoregulation, K⁺/Na⁺ balance

## Abstract

**Background:**

Salt stress disrupts cellular water status, redox homeostasis, and ionic balance, thereby limiting the growth and ornamental performance of *Pyrus calleryana*. This study investigated the physiological mechanisms underlying melatonin-mediated salt tolerance.

**Results:**

Seedlings subjected to 0.45% NaCl were foliar-sprayed with 50–200 µmol·L⁻¹ melatonin. Salt stress inhibited growth, enhanced membrane lipid peroxidation, reduced relative water content and photosynthetic capacity, and disturbed K⁺/Na⁺ ratio homeostasis. Melatonin application alleviated these adverse effects, with 100 µmol·L⁻¹ showing the strongest effect. Melatonin treatment increased proline and soluble protein levels, enhanced SOD, POD, and CAT activities, reduced MDA content and REC, and improved chlorophyll fluorescence and gas exchange parameters.

**Conclusions:**

Comprehensive analysis revealed that the enhanced salt tolerance was associated with coordinated regulation between osmotic adjustment and antioxidant defense systems, which collectively stabilized membrane integrity, maintained cellular water status, and supported photosynthetic performance under salt stress.

## Introduction

Soil salinity is recognized as a significant constraint on ornamental plant cultivation. Under saline conditions, plants first encounter water deficit and ionic disequilibrium, which together disturb normal physiological processes [1, 2]. Salinity also disrupts intracellular redox balance, resulting in oxidative stress that compromises membrane stability and cellular metabolism [3, 4].

Saline soils are widely distributed worldwide, covering approximately 1.1 × 10⁹ hm² and continuing to expand. In China, there are about 3.67 × 10⁷ hm² of saline soil, which is extensive, diverse, and widely distributed across different climatic zones. Among them, soil salinization in arid and semi-arid regions is particularly severe [5, 6]. Given the growing risk of salt stress, it is imperative to investigate practical methods for improving ornamental plants’ ability to withstand salt.

*Pyrus calleryana* Decne. produces clusters of pure white blooms, yellow-brown spherical fruit, and vibrant red foliage in the fall. The attractive flowers, foliage, and fruiting traits of this species make it an outstanding decorative plant [7, 8]. At the same time, it exhibits vigorous growth and strong adaptability. It can tolerate drought and poor soil conditions, and shows low edaphic requirements. Therefore, it has great potential in urban greening. However, it is relatively sensitive to soil salinity. Salt stress can reduce its ornamental value and even affect its normal growth and development [9].

While traditional breeding methods take a long time to improve salt tolerance, molecular breeding approaches are complex and difficult to integrate with existing excellent traits. The use of plant hormones to increase salt tolerance, in contrast, provides a faster, simpler, and less expensive option than traditional breeding, avoiding the drawn-out processes and complicated molecular methods [10].

Plant hormones are crucial regulatory factors during the growth and development of plants, and they mediate responses to both biotic and abiotic stresses [11–13]. Accumulating evidence indicates that exogenous addition of plant hormones can alleviate the harm caused by adverse conditions to plants [14, 15]. Among them, melatonin (a type of indoleamine), which is widely present in animals and plants, significantly influences plant growth, development, and stress responses, including salinity stress. Exogenous melatonin application has been shown to enhance salinity resistance in soybean plants by elevating the transcription levels of antioxidant enzymes and enhancing photosynthetic capacity [16]. It has also been reported to improve salinity resilience in cotton through regulation of oxidative metabolism and elevating the levels of osmotic regulatory chemicals [17]. The same is true for sugar beets, where exogenous melatonin increases H⁺-ATPase activity, maintains K^+^/Na^+^ homeostasis stable, promotes root growth, and maintains chlorophyll content and photosystem II efficiency in saline environments [18]. All of these results point to the fact that melatonin coordinates multiple physiological processes, including oxidative regulation, water balance, ionic equilibrium, and photochemical performance, hence enhancing plant salt tolerance. These findings provide a physiological basis for the application of melatonin in saline environments.

Currently, melatonin has gradually become an important focus in the research of plant growth regulators. Nonetheless, most previous studies have focused on model plants or herbaceous species. Meanwhile, previous related studies have primarily described isolated physiological responses, with limited attention to the coordinated regulation among multiple physiological systems. However, we hypothesized that exogenous melatonin not only affects individual physiological parameters, but also enhances the salt tolerance of *P. calleryana* through coordinated regulation among multiple systems. Therefore, this study aimed to: (1) clarify the dose effect of exogenous melatonin on the salt tolerance of *P. calleryana*; (2) reveal the coordinated response mechanisms underlying changes in different physiological parameters. This study provides new insights into the system-level regulation of salt tolerance in ornamental woody plants by melatonin.

## Materials and methods

### Plant material

The study was carried out in Baoding City, Hebei Province, China (38.827222°N, 115.453389°E) at the Laboratory of Plant Physiology and Biochemistry, College of Landscape and Tourism, Hebei Agricultural University. *P. calleryana* specimens used in the study were sourced from Lvze Family Farm, Hengshui City, Hebei Province.

### Experimental setup

One-year-old seed-grown *P. calleryana* seedlings were cultivated in plastic pots with a diameter of 50 cm, filled with a mixed substrate of garden soil and sand (v/v = 3:1), and maintained in a greenhouse. The greenhouse conditions were set at 25 ± 2 °C, with a photoperiod of 14 h light/10 h dark and a relative humidity of 60–70%. Preliminary experiments showed that a soil salinity level of 0.45% (w/w) NaCl significantly inhibited the normal growth of *P. calleryana* seedlings; therefore, this salinity level was selected for the salt stress treatment in this study.

After 70 days of cultivation, uniform seedlings were assigned to six treatments using a completely randomized design: CK (distilled water); T1 (0.45% NaCl); T2 (50 µmol·L⁻¹ melatonin + 0.45% NaCl); T3 (100 µmol·L⁻¹ melatonin + 0.45% NaCl); T4 (150 µmol·L⁻¹ melatonin + 0.45% NaCl); and T5 (200 µmol·L⁻¹ melatonin + 0.45% NaCl).

Seedlings in the T2–T5 groups were sprayed daily at 19:00 with 20 ml of the corresponding solutions for five consecutive days. Spraying was continued until the solution began to drip from the leaf surface. CK plants received the same volume of distilled water. After melatonin application, seedlings were subjected to salt stress treatment corresponding to a soil NaCl content of 0.45% (w/w). The CK group was irrigated with the same amount of distilled water. During the entire experiment, salt stress was maintained by watering each pot with 200 mL of 0.45% (w/v) NaCl solution every two days. The CK group was irrigated with an equal amount of distilled water.

Each treatment included three biological replicates (*n* = 3), with each seedling treated as an independent biological replicate. For physiological and biochemical analyses, three technical replicates were performed for each biological replicate for Pro, SP, REC, MDA, antioxidant enzyme activities, and photosynthetic pigment contents. Photosynthetic gas exchange and chlorophyll fluorescence parameters were measured three times per leaf, and the average values were used for statistical analysis. At 10:00 a.m. on days 5, 10, 15, and 20 following salt treatment, fully expanded functional leaves were gathered from the same position of each seedling. Samples were kept at -80 °C for further physiological analyses after being instantly frozen in liquid nitrogen.

### Measurement of physiological and biochemical indicators

Plant growth under different treatments was quantified using plant height increment and ground diameter increment as evaluation indices. Plant height increment was measured using a steel ruler, while ground diameter increment was determined with a vernier caliper.

Relative water content (RWC) was calculated based on fresh weight (W₁), turgid weight (W₂), and dry weight (W₃) as [19]:

$$\mathrm{RWC}\;(\%)=\frac{{W}_{1}-{W}_{3}}{{W}_{2}-{W}_{3}}\times\:100$$

Relative electrical conductivity (REC) and malondialdehyde (MDA) content were determined according to the procedure described by Castelli and Lutts [20, 21].

Proline (Pro) concentration was determined via the ninhydrin assay [22], and soluble protein (SP) content was measured by the Coomassie Brilliant Blue G-250 assay at 595 nm [23].

Song et al. served as the basis for the analysis of antioxidant enzyme activity [24]. Fresh tissue (0.5 g) was homogenized in pre-cooled phosphate buffer, and the volume was adjusted to 5 mL. The supernatant was recovered as a crude enzyme solution and kept on ice following centrifugation at 10,000 rpm for 20 min. SOD activity was quantified following the NBT assay protocol, the guaiacol method was used to measure POD, and CAT activity was determined using the potassium permanganate titration method. SOD was measured at a wavelength of 560 nm, and POD was measured at 470 nm.

Photosynthetic pigments were extracted in 95% ethanol in darkness for 24 h. Absorbance was recorded at 663, 645, and 470 nm, and pigment concentrations were calculated accordingly [25].

A Li-6400 portable photosynthesis analyzer (LI-COR Biosciences, USA) was used to measure *P*_*n*_, *G*_*s*_, *T*_*r*_, and *C*_*i*_ on clear days between 9:00 and 11:00. Following the manufacturer’s instructions, fluorescence parameters (Fv/Fo and Fv/Fm) were measured on identical leaves using a FluorPen meter in identical environmental conditions.

For ion analysis, samples were collected separately from leaves, stems, and roots at day 20 after salt treatment. Potassium and sodium contents were quantified by flame photometry, and the K⁺/Na⁺ ratio was subsequently calculated [26].

### Statistical analysis

All raw data were organized in Microsoft Excel, and subsequent statistical analyses and figure generation were conducted using SPSS 26.0, SPSSAU (https://spssau.com) and Origin 2021.

Data are expressed as mean ± SD of three biological replicates (*n* = 3). Differences among treatments were determined using Duncan’s multiple range test at the 0.05 probability level (SPSS 26.0). Principal component analyses (PCA) and Pearson correlation analysis were conducted using Origin 2021 software. All variables were standardized before PCA to mitigate the effects of differing units and scales.

A membership function approach was applied to assess the overall physiological status of *P. calleryana* seedlings under various treatments. The membership function value (µ_ij_) was calculated as:

$$\:{\mu\:}_{ij}=\frac{{X}_{ij}-{X}_{\mathrm{m}\mathrm{i}\mathrm{n}}}{{X}_{\mathrm{m}\mathrm{a}\mathrm{x}}-{X}_{\mathrm{m}\mathrm{i}\mathrm{n}}}$$

Grey relational analysis and coupling coordination analysis were performed using SPSSAU. Grey relational analysis (GRA) was applied to evaluate the association between individual indicators and the comprehensive response under salt stress. The measured RWC values were defined as the reference sequence, whereas the measured values of other physiological indicators were used as comparison sequences, because RWC is a widely used indicator reflecting plant water status and overall physiological responses under salt stress and is closely associated with osmotic adjustment, membrane stability, and photosynthetic performance.

To further quantify the interaction between osmotic regulation and antioxidant defense systems, a coupling coordination model was established. All subsystem indices were normalized prior to calculation, and equal weights were assigned to each subsystem. The calculated D values were interpreted according to widely used classification standards reported in related studies [27].

Variation partitioning analysis (VPA) was performed to explore the relative contributions of salt stress and melatonin-associated treatment effects to different physiological trait groups under the experimental conditions. Because melatonin treatments were only applied under salt stress conditions, the VPA results should be interpreted as exploratory rather than strictly independent effects.

## Results

### Growth performance under melatonin treatment during salt stress

**Fig. 1:**
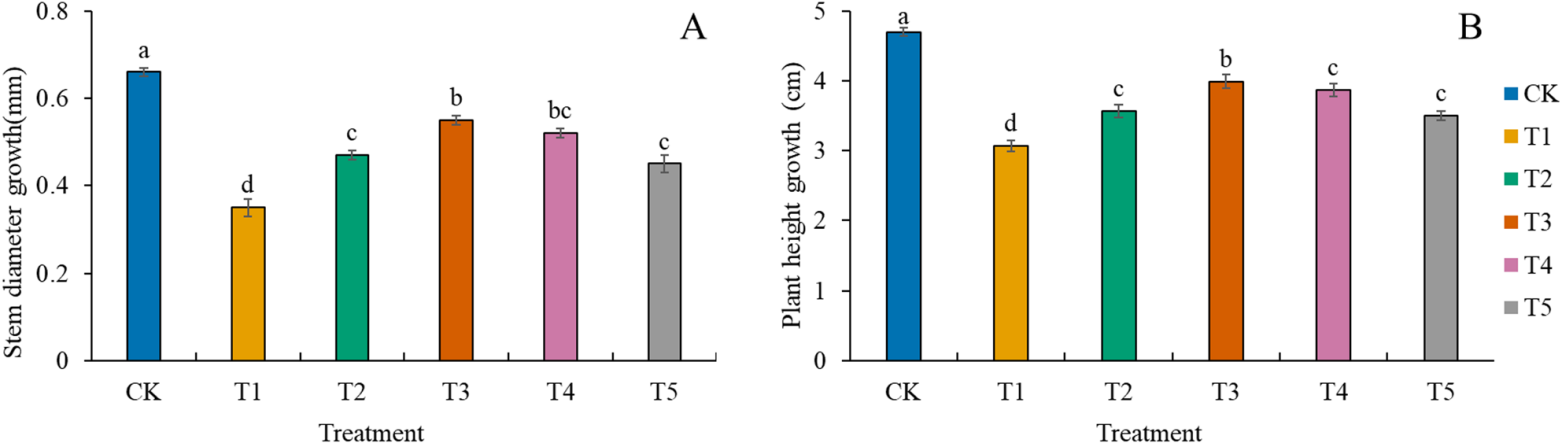
Effects of different treatments on stem diameter growth (**A**) and plant height growth (**B**). Values are presented as mean ± SD (*n* = 3). Different letters indicate significant differences at *p* < 0.05. CK: distilled water; T1: 0.45% NaCl; T2: 50 µmol·L⁻¹ melatonin + 0.45% NaCl; T3: 100 µmol·L⁻¹ melatonin + 0.45% NaCl; T4: 150 µmol·L⁻¹ melatonin + 0.45% NaCl; T5: 200 µmol·L⁻¹ melatonin + 0.45% NaCl

In comparison to the blank control (CK), the salt stress treatment (T1) markedly diminished both stem diameter and plant height growth, with reductions of 46.97% and 34.68%, respectively (Fig. 1). Compared with T1, treatments T2-T5 increased the stem diameter by 34.29%, 57.14%, 48.57%, and 28.57%, respectively. For plant height, treatments T2-T5 increased it by 16.29%, 30.82%, 26.06%, and 14.01%, respectively. Plant height and stem diameter showed the greatest increase under the 100 µmol·L⁻¹ melatonin treatment.

### Leaf water status under melatonin treatment during salt stress

**Fig. 2:**
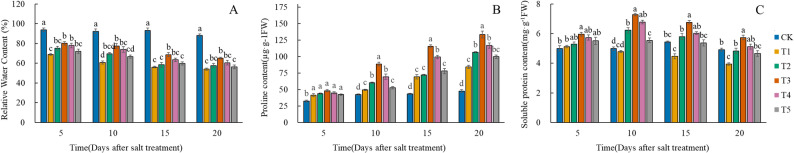
Effects of different treatments on plant relative water content (**A**), proline (**B**) content, and soluble protein (**C**). Values are presented as mean ± SD (*n* = 3). Different letters indicate significant differences at *p* < 0.05. CK: distilled water; T1: 0.45% NaCl; T2: 50 µmol·L⁻¹ melatonin + 0.45% NaCl; T3: 100 µmol·L⁻¹ melatonin + 0.45% NaCl; T4: 150 µmol·L⁻¹ melatonin + 0.45% NaCl; T5: 200 µmol·L⁻¹ melatonin + 0.45% NaCl

In the control group (CK), the RWC of leaves remained largely constant throughout the experiment, as shown in Fig. 2A, whereas salt stress significantly reduced RWC. After exogenous melatonin treatment, leaf RWC increased to varying degrees. With rising melatonin levels, the improvement in RWC first increased before declining; the most significant improvement was seen with T3 treatment. At 20 d, leaf RWC in the T3 treatment was 20.64% higher than in the T1 treatment.

### Osmotic adjustment under melatonin treatment during salt stress

Compared with the control (CK), under salt stress (T1), the proline (Pro) content in leaves was significantly increased at all sampling time points (Fig. 2B), while the soluble protein (SP) content was significantly decreased at 15 and 20 days (Fig. 2C). Compared with T1, the Pro content under the T2–T4 treatments was significantly increased at 10, 15, and 20 days. At day 20, the Pro content under the T2, T3, and T4 treatments increased by 26.36%, 58.28%, and 38.41%, respectively, compared with T1.

At 10–20 days, the SP content under the T2–T4 treatments was higher than that under T1; however, no significant difference in SP content was observed between the T5 and T1 treatments at 5–20 days. Among all treatments, the Pro and SP levels under the T3 treatment were consistently higher than those of the other melatonin treatments.

### Antioxidant defense under melatonin treatment during salt stress

**Fig. 3:**
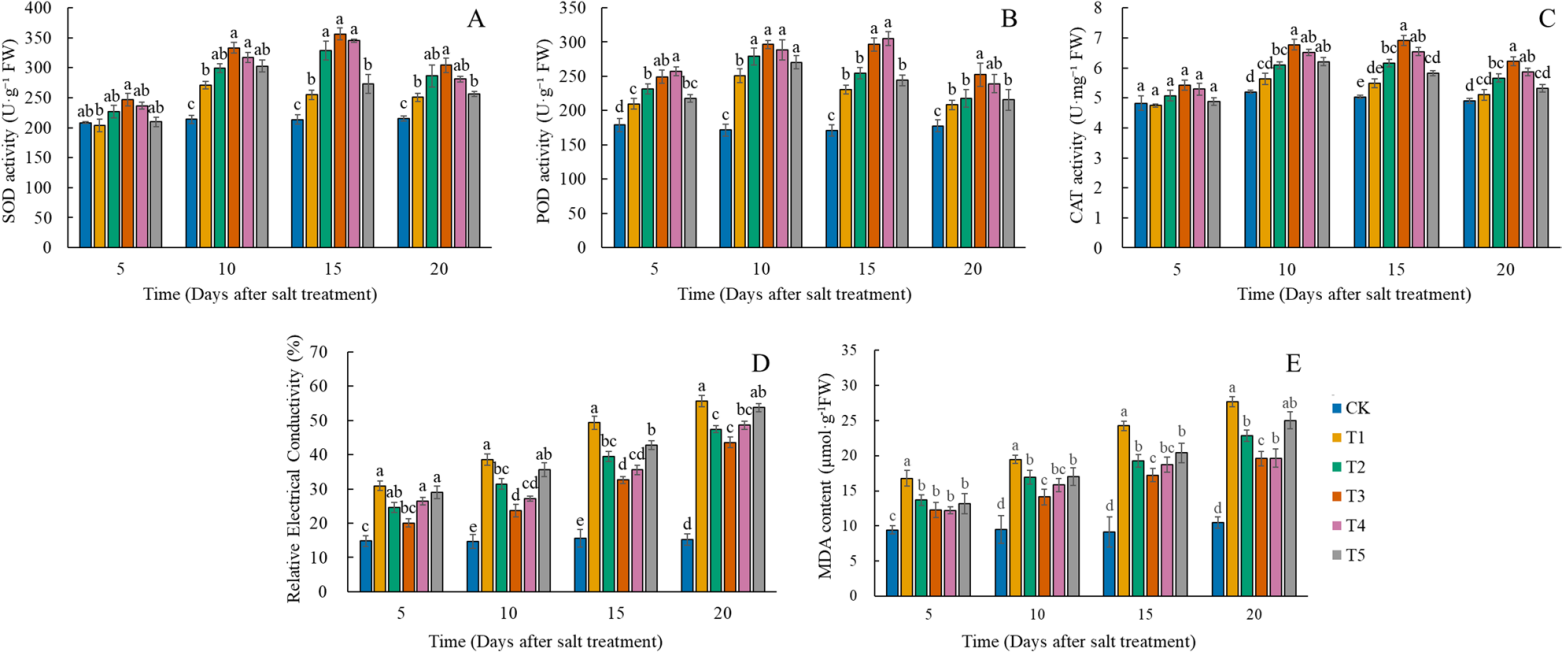
Effects of different treatments on plant SOD activity (**A**), POD activity (**B**), CAT activity (**C**), Relative electrical conductivity (**D**), and MDA (**E**) content. Values are presented as mean ± SD (*n* = 3). Different letters indicate significant differences at *p* < 0.05. CK: distilled water; T1: 0.45% NaCl; T2: 50 µmol·L⁻¹ melatonin + 0.45% NaCl; T3: 100 µmol·L⁻¹ melatonin + 0.45% NaCl; T4: 150 µmol·L⁻¹ melatonin + 0.45% NaCl; T5: 200 µmol·L⁻¹ melatonin + 0.45% NaCl

As shown in Fig. 3, under the T1–T5 treatments, the activities of the three antioxidant enzymes gradually increased from 5 to 15 days and then decreased at 20 days. Compared with CK, under the T1 treatment, SOD, POD, and CAT activities increased by 19.72%, 34.89%, and 8.92%, respectively, at day 15, and the SOD and POD activities under T1 were significantly higher than those of CK. After the application of exogenous melatonin, the activities of the three antioxidant enzymes were further increased compared with T1. Compared with the T1 treatment, the SOD activity of *P. calleryana* seedlings under the T2–T5 treatments at day 15 increased by 28.89%, 39.74%, 35.29%, and 7.06%, respectively (Fig. 3A). The POD activity increased by 10.40%, 28.47%, 32.23%, and 5.92%, respectively (Fig. 3B). The CAT activity increased by 12.20%, 26.05%, 19.13%, and 6.01%, respectively, compared with T1 (Fig. 3C). Among all treatments, the T3 treatment exhibited the highest antioxidant enzyme activities at day 15.

### Membrane stability under melatonin treatment during salt stress

As shown in Fig. 3, compared with the control (CK), under salt stress (T1), REC and MDA contents were significantly increased at all sampling time points. In addition, from 5 to 15 days, the T1–T5 treatments showed a clear increasing trend. At day 5, compared with CK, REC and MDA contents under T1 increased by 109.00% and 77.92%, respectively. By day 20, REC and MDA contents under T1 further increased to 266.53% and 164.88%, respectively, compared with CK.

Compared with T1, at day 20, the relative electrical conductivity under the T2–T5 treatments decreased by 14.77%, 21.69%, 12.69%, and 3.41%, respectively, while MDA content decreased by 17.41%, 29.26%, 29.44%, and 9.57%, respectively. Among all treatments, the T3 treatment exhibited the lowest REC and MDA values at day 20.

### Gas exchange parameters under melatonin treatment during salt stress

**Fig. 4:**
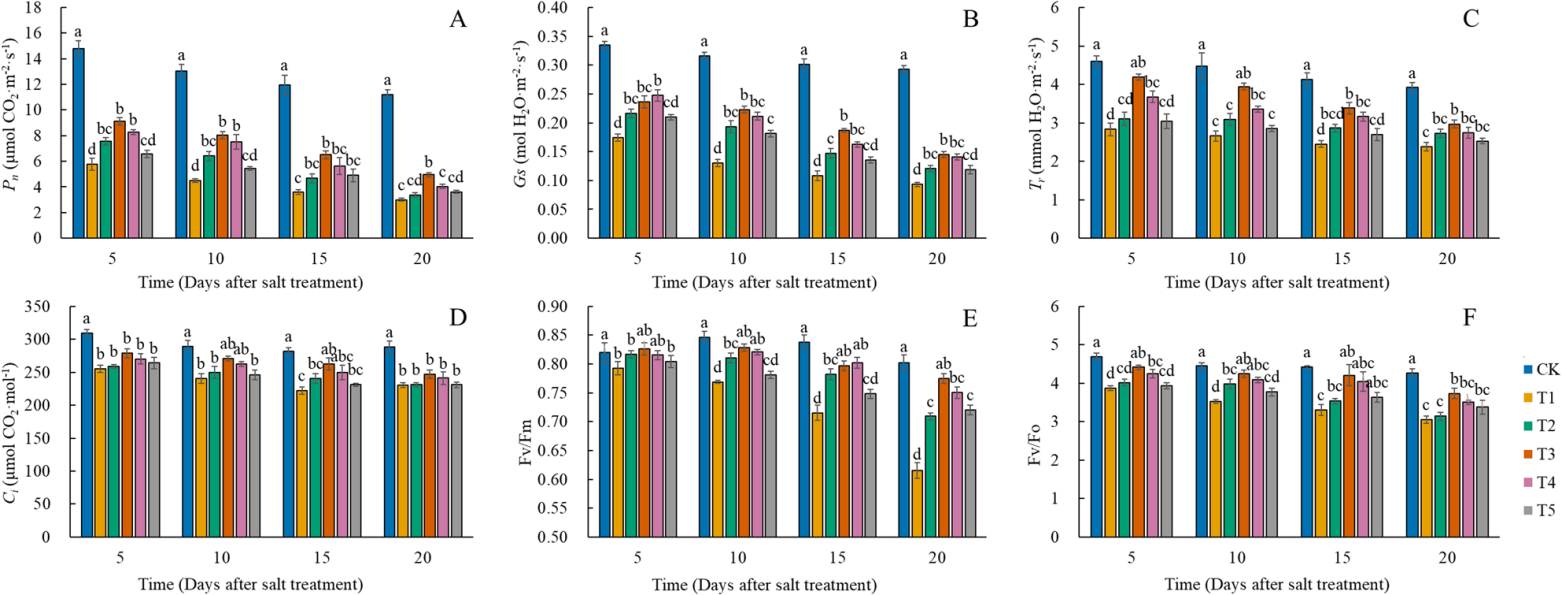
Effects of different treatments on plant photosynthetic parameters *P*_*n*_ (**A**), *G*_*s*_ (**B**), *T*_*r*_ (**C**), *C*_*i*_ (**D**), Fv/Fm (**E**), and Fv/Fo (**F**). Values are presented as mean ± SD (*n* = 3). Different letters indicate significant differences at *p* < 0.05. CK: distilled water; T1: 0.45% NaCl; T2: 50 µmol·L⁻¹ melatonin + 0.45% NaCl; T3: 100 µmol·L⁻¹ melatonin + 0.45% NaCl; T4: 150 µmol·L⁻¹ melatonin + 0.45% NaCl; T5: 200 µmol·L⁻¹ melatonin + 0.45% NaCl

As shown in Fig. 4, under the T1–T5 treatments, *P*_*n*_, *G*_*s*_, *T*_*r*_, and *C*_*i*_ of *P. calleryana* leaves showed a gradual decreasing trend with the extension of the treatment time. By day 20, *P*_*n*_, *G*_*s*_, *T*_*r*_, and *Ci* under the T1 treatment were significantly reduced by 73.26%, 68.14%, 39.27%, and 19.96%, respectively, compared with CK.

At day 20, *P*_*n*_, *G*_*s*_, and *T*_*r*_ under the T3 treatment increased by 65.00%, 55.52%, and 24.37%, respectively, compared with T1, and were significantly higher than those under T1. At this time, there was no significant difference in *C*_*i*_ between the T3 and T1 treatments. Among all treatments, T3 exhibited the highest *P*_*n*_, *G*_*s*_, and *T*_*r*_ values at day 20.

### Chlorophyll fluorescence parameters under melatonin treatment during salt stress

The Fv/Fm and Fv/Fo values of the leaves in the control group (CK) stayed comparatively constant over the course of the experiment, as illustrated in Fig. 4. Compared with CK, Fv/Fm under salt stress (T1) exhibited a continuous decline with increasing treatment duration, with a more pronounced decrease observed at later stages. At day 20, the Fv/Fm value under T1 was significantly reduced by 23.24% relative to CK. Following the application of exogenous melatonin, Fv/Fm values increased to varying extents compared with T1, among which the T3 treatment maintained relatively higher levels at all time points. At day 20, Fv/Fm under T3 was significantly increased by 25.93% compared with T1.

The variation trend of Fv/Fo was generally consistent with that of Fv/Fm. Compared with the control (CK), salt stress (T1) led to a significant decrease in Fv/Fo, and the magnitude of decline increased with prolonged treatment duration. Among all the treatments, the T1 treatment showed the most significant decline, with its Fv/Fo at 20 days being significantly reduced by 28.53% compared to CK. Melatonin treatment substantially mitigated the suppressive impact of salinity on Fv/Fo, and the T3 treatment consistently showed higher Fv/Fo values than the other stress treatments. At 20 d, Fv/Fo under T3 increased by approximately 22.50% compared with T1.

### Photosynthetic pigment content under melatonin treatment during salt stress

**Fig. 5:**
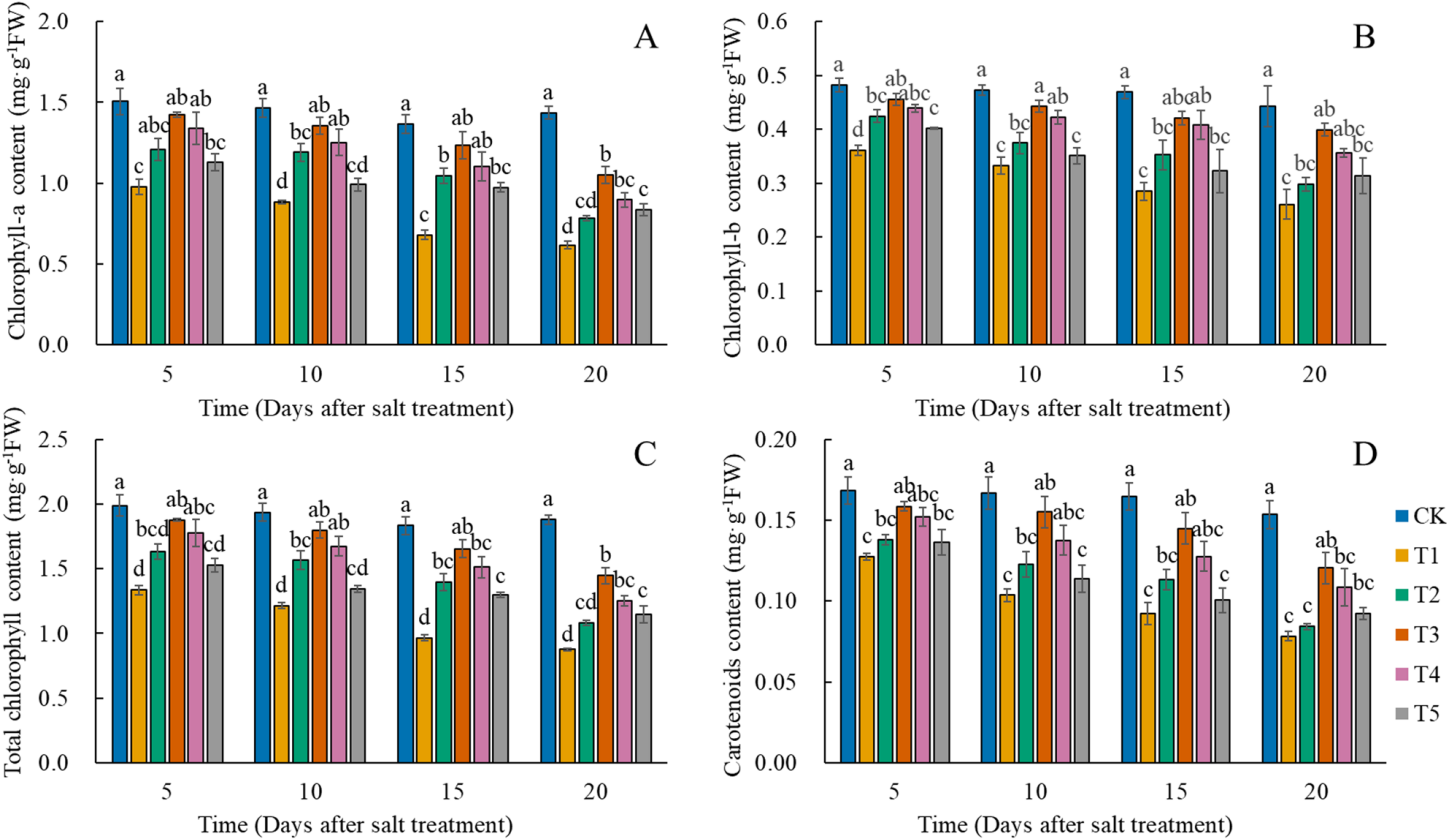
Effects of different treatments on the content of photosynthetic pigments chlorophyll a (**A**), chlorophyll b (**B**), total chlorophyll (**C**), and carotenoids (**D**) in plants. Values are presented as mean ± SD (*n* = 3). Different letters indicate significant differences at *p* < 0.05. CK: distilled water; T1: 0.45% NaCl; T2: 50 µmol·L⁻¹ melatonin + 0.45% NaCl; T3: 100 µmol·L⁻¹ melatonin + 0.45% NaCl; T4: 150 µmol·L⁻¹ melatonin + 0.45% NaCl; T5: 200 µmol·L⁻¹ melatonin + 0.45% NaCl

Compared with the control (CK), under salt stress (T1), the contents of Chl a, Chl b, total chlorophyll, and Car were significantly decreased at all sampling time points, and the pigment contents under T1 gradually declined from 5 to 20 days (Fig. 5). At day 20, compared with CK, Chl a, Chl b, total chlorophyll, and Car under T1 decreased by 41.23%, 53.39%, 56.94%, and 49.06%, respectively.

Compared with T1, after the application of exogenous melatonin, the accumulation of photosynthetic pigments in *P. calleryana* leaves increased (Fig. 5). Among all treatments, T3 exhibited the highest pigment contents at day 20. Under the T3 treatment at day 20, carotenoid, total chlorophyll, chlorophyll a, and chlorophyll b contents were significantly increased by 69.35%, 51.42%, 66.17%, and 53.90%, respectively, compared with T1.

### Ionic homeostasis under melatonin treatment during salt stress

**Fig. 6:**
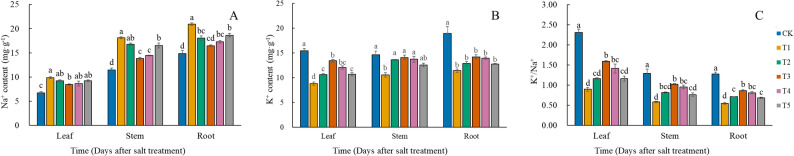
Effects of different treatments on Na⁺ content (**A**), K⁺ content (**B**), and K⁺/Na⁺ ratio (**C**) in leaves, stems, and roots of *P. calleryana* at day 20 after salt treatment. Values are presented as mean ± SD (*n* = 3). Different letters indicate significant differences at *p* < 0.05. CK: distilled water; T1: 0.45% NaCl; T2: 50 µmol·L⁻¹ melatonin + 0.45% NaCl; T3: 100 µmol·L⁻¹ melatonin + 0.45% NaCl; T4: 150 µmol·L⁻¹ melatonin + 0.45% NaCl; T5: 200 µmol·L⁻¹ melatonin + 0.45% NaCl

Compared with the control (CK), under salt stress (T1), Na⁺ content in leaves, stems, and roots was significantly increased, whereas K⁺ content and the K⁺/Na⁺ ratio decreased (Fig. 6). Na^+^ accumulation in leaves, stems, and roots was significantly reduced after exogenous melatonin was applied, as compared to the T1 treatment. This was followed by increases in K^+^ levels and K^+^/Na^+^ ratios. Compared with T1, Na⁺ content under T3 decreased by 14.02%, 23.98%, and 21.24% in leaves, stems, and roots, respectively. Correspondingly, K⁺ content increased by 52.45%, 33.50%, and 24.30% in leaves, stems, and roots, respectively, and the K⁺/Na⁺ ratios were significantly enhanced by 76.67%, 75.86%, and 56.36%, respectively.

### Integrated physiological evaluation under melatonin treatment during salt stress

**Fig. 7:**
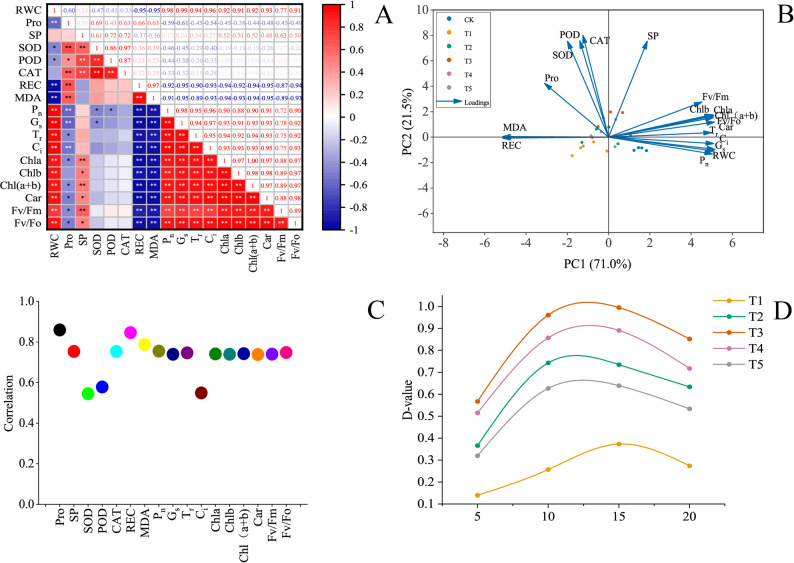
Correlation analysis among physiological indicators (**A**), where * and ** indicate significance at *p* < 0.05 and *p* < 0.01, respectively; PCA of different physiological indicators (**B**); GRA of exogenous melatonin and salt stress (**C**); and Coupling coordination analysis of the antioxidant enzyme activity and osmotic regulatory substances (**D**). RWC: Relative water content; Pro: Proline; SP: Soluble protein; REC: Relative electrical conductivity; MDA: Malondialdehyde; SOD: Superoxide dismutase; POD: Peroxidase; CAT: Catalase; *P*_*n*_: Net photosynthetic rate; *G*_*s*_: Stomatal conductance; *T*_*r*_: Transpiration rate; *C*_*i*_: Intercellular CO₂ concentration; Chla: Chlorophyll a; Chlb: Chlorophyll b, Chl (a + b): Total chlorophyll; Car: Carotenoids

RWC had a significant and positive correlation with photosynthetic pigment content (Chla, Chlb, and Car) and chlorophyll fluorescence parameters (Fv/Fm and Fv/Fo), according to correlation analysis. However, it had a significant and negative correlation with indicators of membrane damage, specifically REC and MDA (Fig. 7A).

Pro and SP were significantly and positively correlated with antioxidant enzyme activities (SOD, POD, and CAT), while antioxidant enzyme activities were significantly and negatively correlated with REC and MDA (Fig. 7A).

Principal component analysis (PCA) was applied to all recorded variables in order to decrease multicollinearity among various physiological markers. With a cumulative contribution of 92.5%, the two principal components, PC1 and PC2, which were extracted, explained 71.0% and 21.5% of the entire variance, respectively (Fig. 7B).

In PC1, photosynthetic pigment contents and related photosynthetic parameters, together with water status and gas exchange–related variables (*G*_*s*_, *C*_*i*_), exhibited high positive loadings. In contrast, membrane damage–related indicators, including REC and MDA, showed high negative loadings on PC1 (Fig. 7B). PC2 was mainly characterized by osmotic adjustment (represented by proline) and antioxidant metabolism.

According to grey relational analysis, there were notable variations in grey relational grades between the RWC of leaves and several physiological indicators (Fig. 7C). Among these parameters, proline content showed the highest grey relational grade, followed by REC and MDA content. In contrast, SOD, POD, and Ci exhibited relatively lower grey relational grades.

Coupling coordination analysis showed that on day 5, T3 and T4 exhibited a marginally coordinated state, whereas T1 had the lowest coupling coordination degree, indicating severe maladjustment (Fig. 7D). During 10–15 days, the coordination levels of T3 and T4 treatments significantly improved, reaching good to excellent coordination. In contrast, the coordination degree of T1 treatment remained at a disordered level. By day 20, the differences in coupling coordination degrees among the various treatments further expanded. T3 showed the highest coupling coordination degree, achieving good coordination; T2 and T4 followed, reaching primary coordination levels, while T1 still exhibited a moderately disordered state. Overall, the coordination between the accumulation of osmotic adjustment substances and antioxidant enzyme activity increased with the extension of treatment time, and the differences among the treatments were significant.

**Fig. 8:**
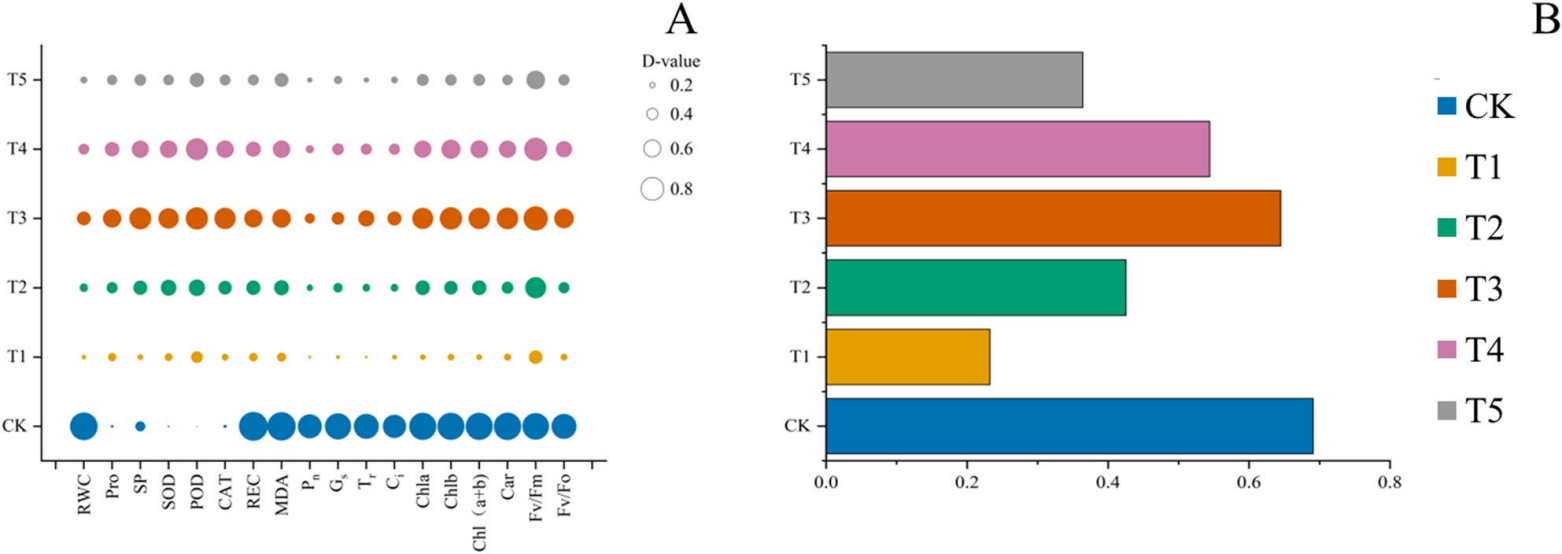
Membership degrees of physiological indicators under different treatments (**A**). Comprehensive evaluation of physiological indicators of salt tolerance in *P. calleryana* under different treatments (**B**). CK: distilled water; T1: 0.45% NaCl; T2: 50 µmol·L⁻¹ melatonin + 0.45% NaCl; T3: 100 µmol·L⁻¹ melatonin + 0.45% NaCl; T4: 150 µmol·L⁻¹ melatonin + 0.45% NaCl; T5: 200 µmol·L⁻¹ melatonin + 0.45% NaCl. Pro: Proline; SP: Soluble protein; REC: Relative electrical conductivity; MDA: Malondialdehyde; SOD: Superoxide dismutase; POD: Peroxidase; CAT: Catalase; *P*_*n*_: Net photosynthetic rate; *G*_*s*_: Stomatal conductance; *T*_*r*_: Transpiration rate; *C*_*i*_: Intercellular CO₂ concentration; Chla: Chlorophyll a; Chlb: Chlorophyll b, Chl (a + b): Total chlorophyll; Car: Carotenoids

The comprehensive physiological performance of plants under salt stress subjected to different melatonin treatments was quantitatively evaluated using the membership function method. The results showed that the distilled water–treated control (CK) exhibited the highest mean membership function value (Fig. 8B). Under salt stress, the membership function value of plants without melatonin application decreased markedly. Membership function values increased compared to T1 with melatonin application, with the highest mean observed under the 100 µmol·L⁻¹ melatonin treatment. The overall performance ranking of treatments was as follows: CK > T3 > T4 > T2 > T5 > T1.

**Fig. 9:**
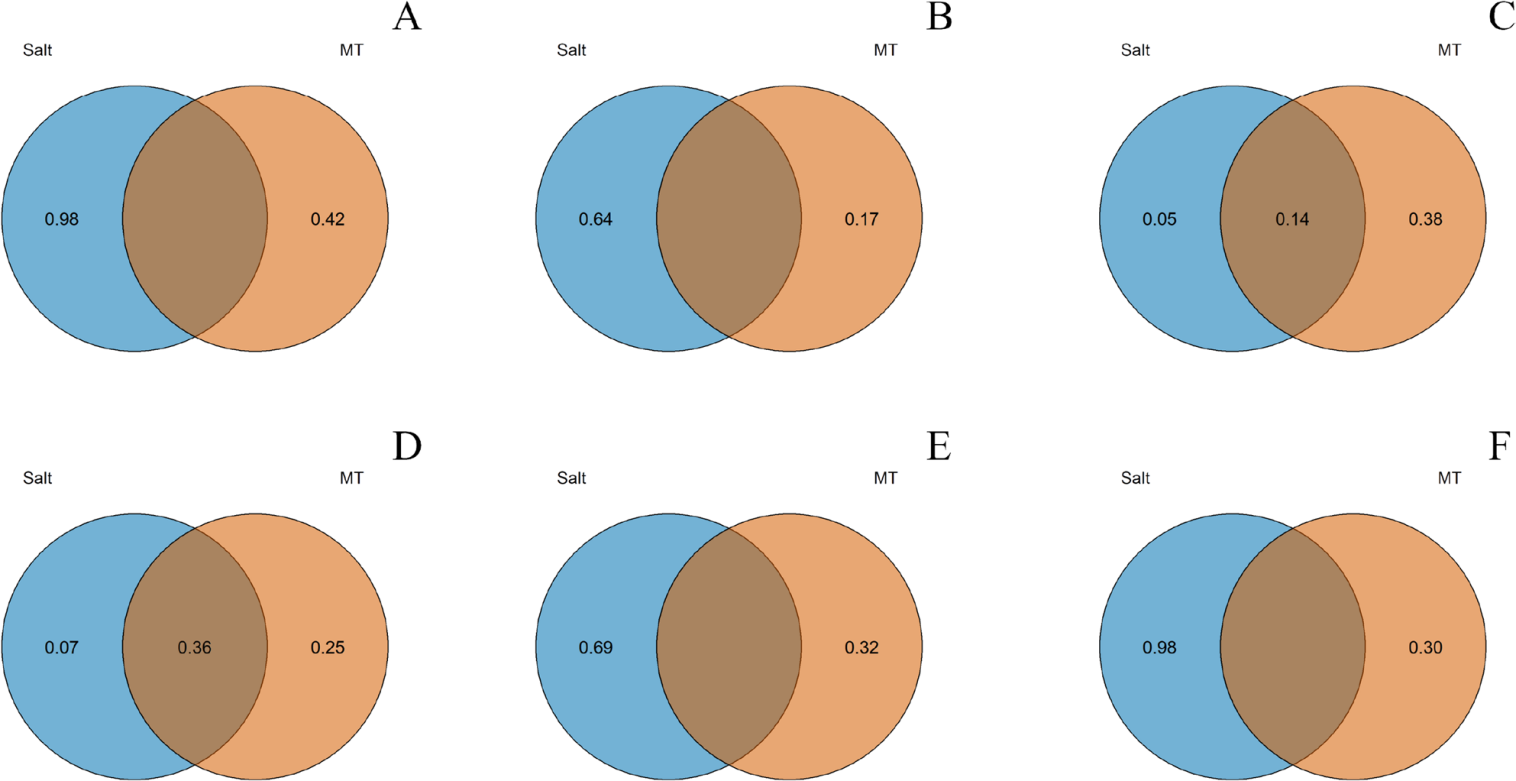
Effects of melatonin and salt stress on physiological traits of *P. calleryana*:(**A**) growth, (**B**) membrane system, (**C**) osmotic adjustment system, (**D**) antioxidant enzymes, (**E**) photosynthesis, and (**F**) ion homeostasis. Values below 0 are not shown

Variation partitioning analysis further quantified the relative contributions of salt stress and melatonin to different groups of physiological traits (Fig. 9). Traits related to growth, membrane integrity, and ion homeostasis were mainly explained by salt stress, whereas the contribution of melatonin treatment was relatively low.

Under salt stress conditions, melatonin-related treatments showed relatively higher contributions to antioxidant-related traits and osmotic adjustment, explaining 0.25 and 0.38 of the variation, respectively. These trait groups also showed a significant shared influence between melatonin and salt stress, especially in the antioxidant enzyme activity, where their combined contribution was 0.36.

## Discussion

Salt stress markedly suppressed growth and photosynthetic performance in *P. calleryana*. Previous studies have shown that melatonin plays an important role in plant salt tolerance [28, 29]. It reinforces cellular defense capacity, stabilizes membrane structure, moderates dehydration effects, and consequently sustains photosynthetic function and growth under saline conditions [30, 31]. After treatment with exogenous melatonin, the plant height and stem diameter were significantly increased compared to the salt treatment. It is notable that the alleviating effect of exogenous melatonin exhibited a typical dose-dependent pattern, increasing initially and then declining with the increase of its concentration, a pattern similar to that reported in *Ginkgo biloba* seedlings exposed to salinity [32]. This may be because excessive exogenous melatonin treatment saturates its action pathways or may disturb hormonal balance and related physiological regulation processes, resulting in a weakened promoting effect.

In this experiment, under salt stress, proline content in leaves increased compared with the control group. Proline is widely recognized as an important osmotic protective agent under abiotic stress conditions [18, 33]. Its accumulation helps maintain the cell osmotic balance, stabilize protein and membrane structures, and alleviate damage caused by stress [34, 35]. At 5 days, soluble protein content under salt treatment also increased, indicating that short-term stress may promote the synthesis of stress-related proteins to cope with salt stress. However, from 10 to 20 days, with the stress duration increased, salt stress enhanced protease activity and accelerated protein degradation, resulting in a gradual decrease in soluble protein content [36]. Notably, the different time patterns of proline and soluble protein changes suggest differences in their functions: proline continuously accumulates throughout the stress period, indicating its role as a long-term osmoprotectant, whereas soluble protein shows a transient increase, reflecting the metabolic adjustment in response to early stress conditions.

Following melatonin treatment, proline and soluble protein contents increased to varying degrees relative to the salinity-only treatment. The results showed that exogenous melatonin may promote proline accumulation and maintain protein stability under salt stress, thereby contributing to osmotic balance and cellular water potential.

Salt stress induces excessive ROS generation, and the overaccumulation of ROS may lead to membrane damage, protein degradation, and disruption of cellular metabolism, and increased malondialdehyde (MDA) content, thereby affecting normal cellular metabolic functions [37, 38]. Meanwhile, damage to membrane stability can also cause ion leakage and increase REC [39].

Under abiotic stress, melatonin treatment is frequently accompanied by elevated antioxidant enzyme activity and strengthened ROS-scavenging capacity. Melatonin and its metabolites can also directly neutralize ROS because they are endogenous antioxidants [40–43]. Significant increases in SOD and POD activity were observed after salt treatment as compared to CK, indicating the presence of stress-induced antioxidant activation. Relative water content decreased, whereas MDA content and REC increased under salt stress, indicating that oxidative stress exceeded the antioxidant defense capacity, leading to membrane damage. The results showed that although *P. calleryana* reacted to salt stress, the damage exceeded the cellular self-regulation capacity.

The activities of SOD, POD, and CAT in *P. calleryana* leaves were elevated subsequent to exogenous melatonin administration. In comparison to the salt stress treatment, the RWC rose while the MDA and REC in the leaves fell to various degrees. This suggests that under salt stress, melatonin may enhance enzymatic antioxidant responses and contribute to the maintenance of membrane stability under salt stress. Notably, antioxidant enzyme activities decreased after reaching a peak around day 15, which may reflect a transition from short-term adaptive responses to long-term stress-induced damage. Melatonin treatment delayed the downward trend of antioxidant enzyme activity and maintained relatively high enzyme activity in the later stage, indicating that melatonin may help extend the effective defense period and reduces oxidative damage to the cell membrane.

Photosynthetic pigments determine the efficiency of light capture and are sensitive to salinity [44]. Under salt stress, excessive Na⁺ enters the cells, damaging the lipid bilayer structure of the thylakoid membrane, which affects its light-harvesting capacity. Meanwhile, salt stress may impair chlorophyll biosynthesis and disrupt thylakoid membrane structure [45, 46]. Therefore, salt stress often leads to pigment loss and a decrease in the efficiency of photosystem II [47–49]. In agreement with this, the concentration of carotenoids and chlorophyll in *P. calleryana* leaves, together with the chlorophyll fluorescence parameters Fv/Fm and Fv/Fo, were markedly reduced under salt stress in the present study. In contrast to salt treatment alone, exogenous melatonin significantly raised pigment levels and maintained higher Fv/Fm and Fv/Fo values. This may be attributed to the fact that melatonin treatment enhanced the activity of antioxidant enzymes and the content of osmotic regulatory substances, thereby helping maintain membrane stability and alleviating chlorophyll degradation [30]. Additionally, the increase in chlorophyll concentration may have contributed to the enhancement of PSII performance, which is consistent with previous findings [50].

Photosynthetic traits are closely linked to plant productivity and are often used to assess stress responses [51, 52]. In this study, *P*_*n*_, *G*_*s*_, *T*_*r*_, and *C*_*i*_ in *P. calleryana* leaves were drastically decreased by salt stress. This is likely because salt stress reduces leaf relative water content, thereby decreasing stomatal conductance and limiting CO₂ diffusion. Notably, the greater decrease in *P*_*n*_ was observed compared to *C*_*i*_, indicating that non-stomatal limiting factors (such as biochemical or photochemical limitations) also participated in the inhibition of photosynthesis [39]. Following the application of exogenous melatonin, the relative leaf water content rose, the MDA content fell, and *P*_*n*_, *G*_*s*_, and *T*_*r*_ were much higher than those observed in the salt stress treatment. This might be due to the fact that melatonin treatment enhanced antioxidant enzyme activities, alleviated oxidative damage to the photosynthetic apparatus, and at the same time increased the content of osmotic regulatory substances, which helped to improve the water status and stomatal regulation of cells under salt stress. The increase in soluble protein content may reflect an increase in the synthesis of photosynthetic enzymes such as Rubisco, thereby enhancing photosynthetic performance [46].

In a high-salt environment, excessive Na⁺ enters plant cells, disrupting metabolic processes and competitively inhibiting K⁺ uptake and transport, thereby disturbing cellular ionic balance [53]. Therefore, the capacity to maintain K⁺ content is commonly regarded as an important indicator of plant salt tolerance [54, 55]. In this study, salt stress increased the Na⁺ content in different organs of *P. calleryana*, while reducing K⁺ level, resulting in an ion imbalance. This is consistent with the previous research results [56]. Notably, excess Na⁺ was predominantly retained in the roots and stems. This may be attributed to Na⁺ compartmentalization, whereby plants preferentially sequester excess Na⁺ in roots and stems under salt stress, limiting its translocation to leaves and thus protecting photosynthetic tissues from ion toxicity [57–59].

Melatonin may contribute to the maintenance of ionic equilibrium under salinity through its effects on ion transport [60]. Compared with salt stress treatment, melatonin treatment markedly decreased Na⁺ levels in roots and stems, while the Na⁺ content in leaves did not change significantly. K⁺ content showed an opposite trend. The reduction in Na⁺ content in roots and stems may be associated with melatonin-mediated regulation of ion homeostasis, which could enhance ion selectivity [61, 62]. The significant increase in K⁺ content in leaves might be due to melatonin, which significantly reduced MDA content, thereby stabilizing the membrane stability and reducing K⁺ efflux [32]. When treated with 100 µmol·L⁻¹ melatonin, a relatively high K⁺/Na⁺ ratio was observed in different organs, indicating that the effect of melatonin on regulating ion homeostasis was the most obvious at this concentration. In this experiment, plants with a relatively high K⁺/Na⁺ ratio often showed higher RWC, lower REC, lower MDA content, and better photosynthetic performance. These results suggest that melatonin contributes to salt tolerance by maintaining ionic homeostasis, which works together with osmotic and antioxidant regulation to sustain cellular physiological stability.

Salt tolerance cannot be evaluated by a single physiological indicator [63]. To explore the physiological mechanisms underlying the enhancement of salt tolerance in *P. calleryana* by exogenous melatonin, this study employed various analytical methods to analyze the experimental data.

RWC is a key indicator for assessing plant stress tolerance under salt stress [1]. Correlation analysis showed that RWC was significantly positively correlated with photosynthetic parameters and negatively correlated with membrane damage indicators. The positive relationship between RWC and photosynthetic parameters may be attributed to its role in maintaining stomatal opening and CO₂ diffusion, as well as preserving chloroplast structure and enzyme activity. Membrane damage is often associated with ROS accumulation, and the negative correlation between RWC and membrane damage indicators highlights the critical role of plant water status in stabilizing cellular structures and alleviating oxidative stress. PCA indicates that the physiological markers under salt stress are primarily categorized into two modules: structural damage and defensive regulation. Exogenous application of melatonin shifted the sample scores on the principal component factors in a higher direction, indicating that melatonin enhanced defense capacity and improved the integrity of physiological structures. Grey correlation analysis showed that Pro had the strongest correlation with overall physiological performance, followed by REC and MDA. These results suggest that osmotic regulatory substances are key indicators of overall physiological performance. Pro can safeguard enzymes associated with reactive oxygen detoxification and, as an osmotic regulating agent, preserves osmotic equilibrium and ensures the membrane stability [64].

Multivariate analyses (PCA and coupling coordination) consistently indicated that osmotic adjustment and antioxidant defense were closely coordinated under melatonin treatment. Under salt stress, these two systems are often in a relatively unbalanced state. After applying exogenous melatonin, the coordination between the systems significantly improved, especially under the 100 µmol·L⁻¹ melatonin treatment, where the coordination degree reached a favorable level. This synchrony may help reduce membrane lipid peroxidation, while improving photosynthesis and ionic balance [65, 66]. The VPA analysis results revealed the roles of salt stress and melatonin in different functional traits. Among them, salt stress had a relatively high independent contribution to membrane damage, ion imbalance, and growth. This was consistent with the increased phenomena of REC, MDA, Na⁺ accumulation, and growth inhibition under salt stress, indicating that these traits mainly reflected the direct effect of salinity.

Melatonin-associated treatment effects were more strongly associated with antioxidant enzyme activity and osmotic regulation, indicating that melatonin may be more responsive to these physiological processes under salt stress. Antioxidant- and osmotic-related features showed considerable shared variance between salt stress and melatonin, suggesting that the two variables work together to modulate these responses.

Based on the above results, a more integrated understanding of the mechanism by which melatonin regulates salt tolerance in *P. calleryana* can be proposed. Although osmotic regulation and antioxidant defense were initially considered as independent physiological responses, comprehensive analysis suggests that these two systems are functionally interconnected rather than separate. Specifically, increased accumulation of osmotic regulatory substances helps maintain cellular water balance and stabilize protein structures, thereby creating favorable conditions for antioxidant defense. Conversely, enhanced antioxidant capacity can mitigate reactive oxygen species–induced membrane damage, protecting membrane integrity and reducing solute leakage. This interaction contributes to the maintenance of cellular water balance and ion homeostasis, ultimately supporting photosynthetic performance under salt stress.

**Fig. 10:**
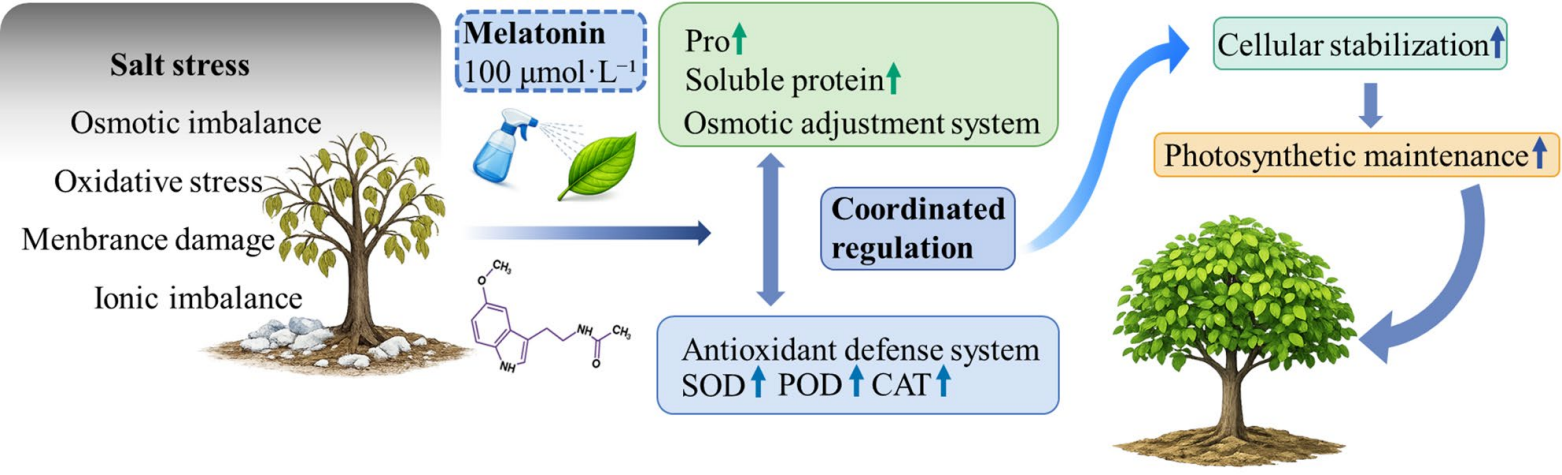
Exogenous melatonin (100 µmol·L⁻¹) enhances salt tolerance in *P. calleryana* by coordinately strengthening osmotic adjustment and antioxidant defense systems, stabilizing membrane integrity and ion homeostasis, improving photosynthesis, and ultimately promoting plant growth under salt stress

Therefore, the regulatory role of melatonin should be understood as a coordinated multi-system response rather than a simple aggregation of isolated physiological regulations. This coordination appears to arise from the dynamic interactions among osmotic adjustment, redox balance, and membrane stability, forming an integrated network that supports the enhancement of salt tolerance (Fig. 10).

Notably, although melatonin generally shows a promotive effect, its efficacy declines at higher concentrations, which is consistent with the results of previous studies. This may be related to disturbances in redox homeostasis and physiological signaling processes caused by excessive melatonin application. Previous studies have suggested that excessive scavenging of reactive oxygen species may partially affect their signaling functions, thereby weakening stress defense responses. In addition, high concentrations of melatonin may interfere with endogenous hormonal balance, including hormone-related regulatory processes reported in previous studies [67, 68]. This phenomenon is consistent with the typical hormone response pattern, where an appropriate dose promotes stress resistance, while an excessive dose leads to a weakened effect or even negative consequences.

Finally, this study was conducted under controlled conditions and mainly focused on physiological responses, without exploring the underlying molecular regulatory mechanisms. Future studies should integrate transcriptomic or genomic approaches and conduct validation under field conditions to further elucidate the regulatory network of melatonin in salt stress tolerance.

## Conclusions

In conclusion, exogenous melatonin treatment maintained cell membrane stability, enhanced antioxidant enzyme activity, improved photosynthetic performance and ionic balance, promoted plant growth, and alleviated salt-induced damage. Comprehensive analysis indicates that the physiological response of *P. calleryana* to salt stress shows a multi-system coordinated regulation, and it is strongly dependent on melatonin concentration. Among all treatment schemes, 100 µmol·L⁻¹ melatonin exhibited the greatest overall improvement in salt tolerance. These findings highlight that melatonin-mediated salt tolerance in *P. calleryana* involves coordinated regulation among multiple physiological systems rather than independent responses of single traits, and provide physiological support for the application of melatonin to enhance salt tolerance in *P. calleryana* under salt stress.

## Data Availability

The datasets used and/or analysed during the current study are available from the corresponding author on reasonable request.
